# Changes in Mood States Are Induced by Smelling Familiar and Exotic Fragrances

**DOI:** 10.3389/fpsyg.2016.01724

**Published:** 2016-11-08

**Authors:** Orly Sarid, Michele Zaccai

**Affiliations:** ^1^Department of Social Work, Ben-Gurion University of the NegevBeer-Sheva, Israel; ^2^Department of Life Sciences, Ben-Gurion University of the NegevBeer-Sheva, Israel

**Keywords:** familiar and exotic fragrances, mood states, adjectives, breathing relaxation

## Abstract

Familiar fragrances usually induce positive mood states and elicit favorable evaluation. Relaxation is also widely thought to improve mood state. Yet experimental evidence on the effect of two different stimuli, fragrance smelling and breathing relaxation, on mood state, and fragrance evaluation is lacking. This study aimed to test (1) the effect of two familiar fragrances, lavender and myrtle, and two exotic fragrances, bergamot and ravensara, on perceived mood states before and after relaxation, (2) the effect of relaxation on perceived mood states for each fragrance, and (3) the effect of relaxation on fragrance evaluation as defined by adjectives. We hypothesized that mood states and assessment of the fragrances would differently be affected both in familiar vs. non-familiar fragrances and also before and after relaxation. Participants (*n* = 127) completed questionnaires on their mood states at baseline (T0). They were then presented with each of the four fragrances separately and asked to report on mood state and to assess the fragrances with adjectives before (T1) and after (T2) breathing relaxation. Analyses of the T0–T1 delta values of mood states by ANOVA repeated measures and *post hoc* comparisons showed that mood states were affected by fragrance smelling with no clear differences observed between familiar and exotic fragrances. The same analyses of T1–T2 values showed no differences in mood state after breathing relaxation and fragrance smelling. Fragrance assessment by adjectives indicated a non-conclusive trend for familiar and exotic fragrances. In sum, mood states induced by the fragrance smelling stimulus (T0–T1) were not changed by the addition of the second stimulus of relaxation (T1–T2), indicating that the former stimulus was stronger than the latter. On the other hand, the cognitive component represented by adjective-based assessment of fragrances was slightly modified by the relaxation stimulus.

## Introduction

Fragrances are known to induce mood and emotional responses in human beings (Bensafi et al., 2002; Herz and Inzlicht, 2002; Jo et al., 2013). Indeed, floral scent may even significantly improve one’s mental or emotional state by alleviating anxiety and depressive symptoms (Burnett et al., 2004; Lemon, 2004). Traditionally, human emotional responses to fragrances have been evaluated by using self-report scales of participants exposed to various fragrances that they are asked to assess in terms of specific sets of adjectives (Chrea et al., 2009; Churchill and Behan, 2010). The results of these self-report scales, however, can be strengthened with physiological measurements.

Previous studies have indeed revealed correlations between the evaluation of fragrances and physiological parameters. For example, Bensafi et al. (2002) found a positive correlation between fragrance evaluations of arousal and skin conductance and a negative correlation between evaluations of pleasantness and heart rate. Similarly, exposure to a fragrance produced by fresh plum flowers induced vigor and decreased depressive mood. In parallel, the same fragrance elicited the activation of the sympathetic nervous system and of the movement, speech, and memory centers in the brain (Jo et al., 2013). In another study, differences in EEG brain waves, particularly the enhancement of the alpha wave activity (8–13 Hz) related to relaxation, were also detected following exposure to fragrances (Churchill and Behan, 2010).

While the measurement of scent-driven changes in physiological parameters is straightforward, the evaluation of the effect that fragrance has on mood states can be complex. For example, Jo et al. (2007) found that plant scents generally considered to be stimulating in fact had a relaxing effect on the prefrontal area of the cerebrum. Other reports have shown that different fragrances can induce different mental responses. Exposure to lavender, for example, led to decreases in the performance of working memory and of tasks requiring attention, whereas rosemary enhanced memory quality but impaired the speed of memory. Overall, rosemary significantly induced alertness compared to control (Moss et al., 2003). Likewise, orange odors reduced anxiety and improved the moods of patients waiting for dental care (Lehrner et al., 2005).

Response to fragrances is also affected by the degree of fragrance familiarity, such that familiar scents induce more positive reactions than unfamiliar ones (Herz, 2010). In this study, which was conducted in Israel, we compared participant reactions to familiar fragrances from two plants endemic to the Mediterranean region, e.g., lavender and myrtle (Muñoz-Bertomeu et al., 2007; Migliore et al., 2012), with their responses to two exotic fragrances, bergamot from Asia (Dugo and Bonaccorsi, 2013) and ravensara from Madagascar (Rakotovao and Randrianjohany, 1996).

The generally relaxing effects of fragrances have been investigated in several studies (e.g., reviewed by Angelucci et al., 2014). Relaxation has been associated with reduced norepinephrine levels, which indicate a decrease in autonomic nervous system (ANS) activity (Hoffman et al., 1982). Similarly, practicing meditation as a means of relaxation was accompanied by decreases in central nervous system (CNS) levels of norepinephrine and cortisol (Esch, 2014). Results from another study showed that relaxation and mindfulness enhanced cerebral blood flow (CBF) in brain areas such as the anterior cingulate cortex, the medial prefrontal cortex and the insula (Tang et al., 2015). Yet to the best of our knowledge, the effect of relaxation on fragrance evaluation has not been investigated in the literature.

In this study, we examined (1) the effects of two familiar and two exotic fragrances on perceived mood states before and after relaxation, (2) the effect of relaxation on the perceived mood states for each fragrance, and (3) the effect of relaxation on fragrance appraisal.

Our first hypothesis was that smelling familiar fragrances would affect mood states more strongly than smelling exotic fragrances. We also hypothesized that relaxation would induce a better overall feeling that, in turn, would be reflected by an increase in positive mood states after fragrance smelling. Finally, we hypothesized that, following relaxation, participants would appraise familiar fragrances with more positive adjectives than they would use to assess exotic scents.

## Materials and Methods

### Study Sample

Participants comprised 127 students studying toward their first or second degree at the Israeli universities of Ben-Gurion University of the Negev, The Hebrew University of Jerusalem and Tel-Aviv University. The single inclusion criterion was age under 30. The data were collected between December 2013 and May 2014. Participants were recruited through advertising, face-to-face, and snowball techniques. A similar data collection method was successfully used in another study among students (Sarid et al., 2012).

All participants provided written informed consent, were told that their participation in the study was voluntary, filled in the questionnaires voluntarily, and did not receive any token for their participation. The entire experiment, which included smelling the fragrances and completing the questionnaires, lasted about 90 min. The study was approved by an ethic committee, which ensures conformation with the regulatory standards of Ben-Gurion University of the Negev.

The sample comprised 37.8% (*n* = 48) men and 62.8% (*n* = 79) women with a mean age of 26.11 (*SD* = 2.72). Regarding family status, 81% (*n* = 103) of the participants were single, and the others were either married or in relationships. The majority of the participants was born in Israel (95.3%, *n* = 121) while the remainder was born in the former Soviet Union. Half of the participants resided in the southern region of Israel, 38% resided in the Tel Aviv area and 12% lived in Jerusalem. Undergraduate students constituted the majority of the participants (93%), the rest of who were in the first year of their graduate studies. Two thirds of the participants belonged to the Faculties of Humanities and Social Sciences, while 11% each were from the Health Sciences, Natural Sciences and Engineering Faculties.

### Procedure

One experimenter was involved in the procedure, which entailed participants self-assessing their mood state and then smelling a fragrance and rating the fragrance with a questionnaire constructed for this study.

Baseline (T0): Upon agreeing to take part in the study, all participants confirmed that they were physically and mentally healthy and that they were not suffering from any disease related to sense of smell. Participants were asked to rate their current mood state (using a structured questionnaire, see below).

T1: Each participant was randomly given a vial filled with an essential oil – lavender, myrtle, bergamot, or ravensara – that was referred to simply as a “fragrance” (purchased from a commercial provider, Urim, Israel). Each participant was provided with an opaque box which held a set of four identical, opaque and indistinguishable vials. The vials were positioned randomly in different parts of the box and not in a row. Participants picked one vial at a time. Following smelling, each vial was transferred to another box. A code at the bottom of the vial and invisible to the participant was recorded by the experimenter. While smelling each vial, participants were instructed to assess the fragrance using the adjectives, each of which was presented with a Likert scale (see structured questionnaire below). This stage lasted 10–15 s, a time span shown to reveal significant differences in emotional responses to fragrances (Pichon et al., 2015). Mood states were then measured again as at baseline. This procedure was repeated four times to ensure that all participants were exposed to each of the four mentioned fragrances.

To avoid the risk of habituation from the effects of over questioning, participants were asked to report their moods at T0 only once, and that same baseline assessment was used for all four fragrances.

#### Intervention

A short breathing relaxation exercise was performed for 3–5 min. Participants were told to inhale through their nose and exhale through their mouth while focusing their attention on their stomach area (Varvogli and Darviri, 2011; Sarid et al., 2012).

T2: The procedure described in T1 was repeated after relaxation.

### Measures

#### Mood States

A 15-item list of mood states was used to measure mood, emotion, and wellness. Respondents were asked to evaluate their mood using a 7-point Likert scale, ranging from “strongly disagree” (1) to “strongly agree” (7). A high score for each mood indicated that this mood was highly sensed. In the current study, we examined each mood state individually and did not sum the items. Mood state measurement was based on studies by Rétiveau et al. (2004) and by Churchill and Behan (2010).

#### Adjectives

An 11-item list (Rétiveau et al., 2004) was used to assess the fragrances on a 7-point Likert scale ranging from “not at all” (1) to “very much” (7). A high score for an adjective indicated that the fragrance strongly elicited responses associated with that adjective.

#### Menstrual Cycle

One question referred to the intake of oral contraception and one question to the timing of the menstrual cycle.

#### Demographic Variables

Age, gender, ethno-cultural origin, marital status and location, area of studies, and academic degree were reported.

### Data Analysis

The experiment was divided into three stages. In the first stage, we calculated the difference between mood state values reported at baseline (T0) and those reported at T1 after smelling the fragrances (Delta T0–T1). Positive or negative differences in mood states indicated that the mood state decreased or increased, respectively, after smelling the fragrance. ANOVA repeated measures were performed on the delta between T0–T1 to test the effects of the fragrances within each of the mood states.

In the second stage, we used ANOVA repeated measures to compare mood state values (Delta T1–T2) based on participant ratings of mood state after they had smelled the same fragrances again. In the third stage fragrance assessment (presented as mean values of the adjectives) (Delta T1–T2) was analyzed using ANOVA repeated measures. All the above analyses were followed by *post hoc* comparisons, corrected for multiple comparisons using Bonferroni correction.

## Results

We analyzed the data while taking into account demographic variables. Statistically non-significant differences (*p* ≤ 0.05) in any parameter of fragrance evaluation were found among age, gender, ethno-cultural origin, marital status and location, and area of studies toward academic degree. Therefore, the data were further analyzed taking into account the entire sample.

**FIGURE 1:**
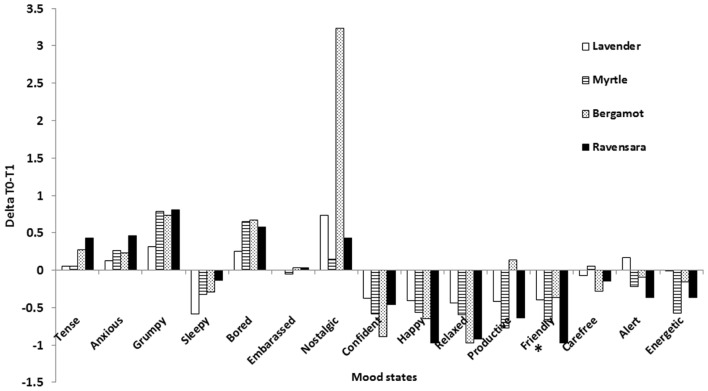
**Mean differences in mood state values between baseline and T1 (Delta T0–T1) for each fragrance.** ^∗^Mood state in which significant differences (at least *p* < 0.05) were recorded among fragrances. See text for details.

To address the effect of fragrance on mood states from baseline to T1, we calculated the delta values between these two time points (**Figure 1**).

To investigate the effect of fragrances within each of the mood states, we performed ANOVA repeated measures on the T0–T1 delta values that, in fact, expressed the moods at T1, since T0 was identical for all fragrances in each mood. This analysis was followed by *post hoc* comparisons. We detected statistically significant differences among fragrances only within the mood state ‘friendly’ (*F* = 4.68; *df* = 3,198; *p* ≤ 0.02). Lavender compared to myrtle and ravensara induced a smaller decrease in this mood state.

Therefore, the first hypothesis – smelling familiar fragrances would affect mood states more strongly than smelling exotic fragrances – was only slightly confirmed.

**FIGURE 2:**
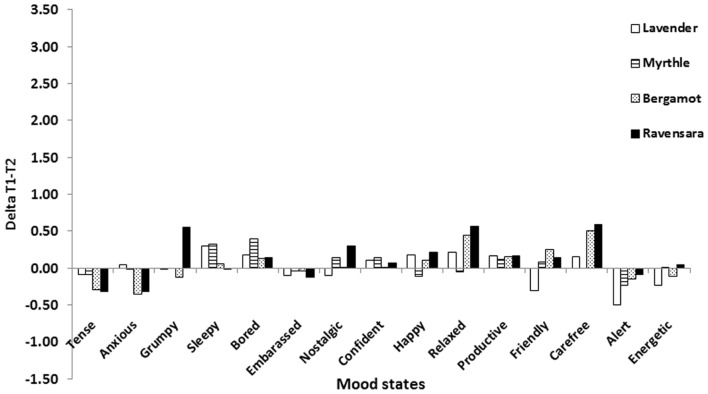
**Mean differences in mood state values between T1 and T2 (Delta T1–T2) for each fragrance**.

The next stage of the analysis involved calculating the differences between mood state values reported at T1 and those reported at T2, after the breathing relaxation intervention and the re-smelling of the fragrances (T1–T2 delta; **Figure 2**). Overall, no differences in mood states were recorded between T1 and T2 after breathing relaxation and fragrance smelling. The effect of fragrances within each mood state was tested by repeated measures ANOVA and was not significant for any of the fragrances (*p* > 0.05, data not shown). Hence, the second hypothesis was not confirmed.

**FIGURE 3:**
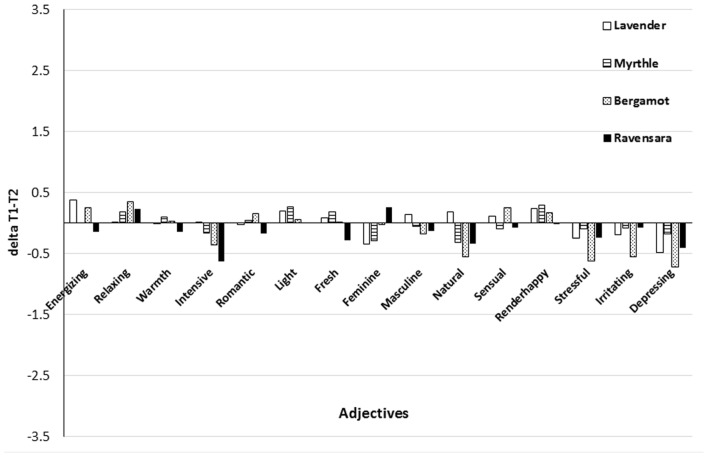
**Mean differences in adjective values between T2 and T1 (Delta T1–T2) for each fragrance**.

Regarding the adjectives used to describe each fragrance, we assumed that familiar fragrances would be more positively appraised than exotic ones following relaxation. Assessment of the familiar fragrance lavender elicited a higher T1–T2 delta than that for the exotic fragrance ravensara for the adjective ‘feminine’ (*F* = 4.35; *df* = 3,198; *p* ≤ 0.01; **Figure 3**). However, as it is unclear whether this adjective reflects positive appraisal, our third hypothesis can be neither confirmed nor rejected.

## Discussion

Fragrances are used to modify mood states and alleviate stress. Similarly, the practice of breathing relaxation generally induces a decrease in anxiety and enhances positive mood states. However, little has been reported about the combined effect of fragrance smelling and breathing relaxation on mood states and fragrance evaluation.

In this study, we examined the effect of two familiar fragrances, lavender and myrtle and two exotic fragrances, bergamot and ravensara, on perceived mood states before and after relaxation. Participants were asked to evaluate their mood states as affected by fragrance and relaxation, and to assess each fragrance according to a list of adjectives.

Our findings (delta T0–T1) demonstrated that fragrances generated responses in mood states that were expressed as an increase or a decrease in a specific mood state. Our data corroborate previous findings showing that fragrances have an impact on mood state (Bensafi et al., 2002; Herz and Inzlicht, 2002; Lehrner et al., 2005; Weber and Heuberger, 2008), an outcome that is allegedly due to the direct connection between the olfactory sense and the limbic system, which regulates emotions in the brain (Lehrner et al., 2005; Jo et al., 2013).

We first hypothesized that smelling familiar fragrances would induce stronger mood state changes than smelling exotic ones. In other words, we expected to find that the absolute values for delta T0–T1 and delta T1–T2 would be higher for familiar than for exotic scents. We found that smelling the familiar fragrance lavender elicited a smaller difference than the exotic ravensara in ‘friendly’ mood state. This minor effect shows that the participants’ mood states were only very slightly, if at all, affected by the familiar or exotic nature of the fragrance. These results differ from a previous report stating that more positive responses are directly correlated to the familiarity of the fragrance (Herz, 2010). It is plausible that the young students who composed our sample have been previously exposed to a variety of fragrances, for example, during their travels, as a consequence of globalization and by the wide availability in Israel of goods from all over the world. Therefore, for these individuals, familiar and exotic fragrances may have become undiscernible.

Our second hypothesis was that relaxation would induce a better feeling in general that, in turn, would be reflected by an increase in positive mood states after fragrance smelling. Our participants were asked to practice deep breathing as a means of relaxation (Varvogli and Darviri, 2011). Contrary to our hypothesis, our results demonstrated that mood states were not modified after relaxation. Positive and negative mood states induced during the first session of fragrance smelling (T0–T1) were conserved during the second session following relaxation (T1–T2). In fact, these results indicate that the fragrance smelling stimulus was stronger in inducing mood states than the relaxation stimulus. It is well accepted that fragrances have a significant impact on emotional responses (Bensafi et al., 2002; Herz and Inzlicht, 2002; Jo et al., 2013). Apparently, the emotional reactions induced by fragrances in our participants during the first smelling session were strong enough to be repeated in the second session. Yet we cannot discern the mechanism that regulates these findings. A possible explanation is that mood states induced by smelling in the first session were preserved and recalled (Sugiyama et al., 2015).

Finally, we hypothesized that, following relaxation, smelling familiar fragrances would be assessed by more positive adjectives than smelling exotic ones. In support of this hypothesis, a significant difference was observed between lavender and ravansara for the adjective ‘feminine.’ However, whether this adjective can be classified as positive or negative is not clear, and therefore, our hypothesis cannot be confirmed. In any case, a clear distinction between familiar and exotic fragrances could not be drawn.

In sum, this study showed that during the fragrance evaluation process, relaxation had a slightly different effect on the adjective-based assessment of the fragrances than it did on mood state evaluation. This could be related to the concept proposed by Scherer (2005), who stated that an emotional response consists of several components, including subjective mood, and cognitive components. Within a specific stimulus, each component may be affected differently (Aue et al., 2007; Grandjean and Scherer, 2008). Consequently, the subjective emotional component (mood state assessment) could have been differently affected than the cognitive component (adjective assessment) by relaxation.

Due to the homogenous nature of the participants, who were students in their late 1920s, the results and conclusions of our study mainly reflect the responses of young adults. Future studies are therefore needed to examine populations that vary by age, such as young children, adolescents, and elderly people. Another possible limitation is that in spite of the random fragrance selection procedure we did not monitor the order of vial selection. Future studies should monitor this aspect as a way to control the impact of a frequently selected fragrance as first choice on the response to the following fragrances. A third possible limitation of our study was the use of specific fragrances. It is plausible that other scents would induce different responses and trends. Future studies should explore the effect of using additional fragrances, including aversive scents, on emotional and cognitive response in the context of breathing relaxation. A fourth limitation was linked to the technique used for relaxation (breathing relaxation). The use of alternative relaxation techniques, such as progressive muscular relaxation (Jacobson, 1938) may induce stronger responses. Finally, the adjective scale that was used in the current study did not yield clear-cut findings. It is possible that a shorter scale presenting fewer adjectives and a more concise Likert scale would yield clearer responses.

## Author Contributions

OS and MZ developed the study design. They conducted the experiments with students and analyzed the data together. Mutual work continued with the writing of the manuscript.

## Conflict of Interest Statement

The authors declare that the research was conducted in the absence of any commercial or financial relationships that could be construed as a potential conflict of interest.
